# Loss of periostin/OSF-2 in ErbB2/Neu-driven tumors results in androgen receptor-positive molecular apocrine-like tumors with reduced Notch1 activity

**DOI:** 10.1186/s13058-014-0513-8

**Published:** 2015-01-16

**Authors:** Roshan Sriram, Vivian Lo, Benjamin Pryce, Lilia Antonova, Alan J Mears, Manijeh Daneshmand, Bruce McKay, Simon J Conway, William J Muller, Luc A Sabourin

**Affiliations:** Department of Cellular and Molecular Medicine, Faculty of Medicine, University of Ottawa, 451 Smyth Road, Ottawa, ON K1H 8M5 Canada; Ottawa Hospital Research Institute, Cancer Therapeutics, 501 Smyth Road, Ottawa, ON K1H 8L6 Canada; Children’s Hospital of Eastern Ontario, Research Institute, 501 Smyth Road, Ottawa, ON K1H8L6 Canada; Department of Biology and Institute of Biochemistry, Carleton University, 1125 Colonel By Drive, Ottawa, ON K1S 5B6 Canada; Developmental Biology and Neonatal Medicine Program, HB Wells Center for Pediatric Research, Indiana University School of Medicine, 705 Riley Hospital Drive, Indianapolis, IN 46202 USA; Department of Biochemistry and Goodman Cancer Research Center, McGill University, 1200 Pine Avenue West, Montreal, QC H3G 1A1 Canada

## Abstract

**Introduction:**

Periostin (Postn) is a secreted cell adhesion protein that activates signaling pathways to promote cancer cell survival, angiogenesis, invasion, and metastasis. Interestingly, Postn is frequently overexpressed in numerous human cancers, including breast, lung, colon, pancreatic, and ovarian cancer.

**Methods:**

Using transgenic mice expressing the Neu oncogene in the mammary epithelium crossed into Postn-deficient animals, we have assessed the effect of Postn gene deletion on Neu-driven mammary tumorigenesis.

**Results:**

Although Postn is exclusively expressed in the stromal fibroblasts of the mammary gland, Postn deletion does not affect mammary gland outgrowth during development or pregnancy. Furthermore, we find that loss of Postn in the mammary epithelium does not alter breast tumor initiation or growth in mouse mammary tumor virus (MMTV)-Neu expressing mice but results in an apocrine-like tumor phenotype. Surprisingly, we find that tumors derived from Postn-null animals express low levels of Notch protein and Hey1 mRNA but increased expression of androgen receptor (AR) and AR target genes. We show that tumor cells derived from wild-type animals do not proliferate when transplanted in a Postn-null environment but that this growth defect is rescued by the overexpression of active Notch or the AR target gene prolactin-induced protein (PIP/GCDFP-15).

**Conclusions:**

Together our data suggest that loss of Postn in an ErbB2/Neu/HER2 overexpression model results in apocrine-like tumors that activate an AR-dependent pathway. This may have important implications for the treatment of breast cancers involving the therapeutic targeting of periostin or Notch signaling.

**Electronic supplementary material:**

The online version of this article (doi:10.1186/s13058-014-0513-8) contains supplementary material, which is available to authorized users.

## Introduction

The epidermal growth factor receptor (EGFR), or HER/ErbB family of receptor tyrosine kinases (RTKs), includes four members, EGFR/HER1/ErbB1, HER2/ErbB2/Neu, HER3/ErbB3, and HER4/ErbB4 playing a role in multiple biological processes such as proliferation, differentiation, migration, and apoptosis [1-3]. Activation of the intracellular kinase domain, through the phosphorylation of carboxyl-terminal tyrosines on HER/ErbB receptors, triggers the association of specific signaling molecules, whose binding initiates downstream signaling events [4].

HER2 (ErbB-2/Neu) is overexpressed in approximately 30% of primary human breast cancers (reviewed [5,6]). HER2 overexpression leads to an aggressive tumor phenotype as high levels of HER2 expression are observed in many invasive human ductal carcinomas, but rarely observed in benign breast disorders. Patients with cancer whose tumors overexpress HER2 receptors tend to have a more metastatic disease with a poor prognosis [5,7].

Transgenic studies have provided direct evidence supporting a role for HER2 in mammary tumorigenesis. Mice expressing a mouse mammary tumor virus (MMTV)-driven activated Neu, the rat homolog of HER2, rapidly develop mammary tumors that histologically resemble human breast carcinomas overexpressing HER2 [8-11].

Periostin (Postn), also designated osteoblast-specific factor-2 (OSF-2), is a disulfide-linked, secreted cell adhesion protein that was originally isolated as an osteoblast- and mesenchyme-specific factor believed to be involved in osteoblast recruitment, attachment, and spreading [12,13]. Postn is primarily expressed in collagen-rich fibrous connective tissues that are subjected to constant mechanical stresses, such as in the periosteum and periodontal ligaments, where it functions in the formation and structural maintenance of bones and teeth [12,13]. Although approximately 14% of Postn-null mice die postnatally before weaning [14], the remaining Postn-deficient mice exhibit severe growth retardation, incisor enamel defects, and an early-onset periodontal disease-like phenotype [14].

Postn binds directly to many extracellular matrix (ECM) proteins such as collagen, fibronectin, tenascin-C, and Postn itself [15]. It also acts as a ligand for several integrins such as α_v_β_3,_ α_6_β_4_ and α_v_β_5_ to mediate cell adhesion, migration and survival [12,15,16]. Interestingly, Postn has also been linked to invasion, cellular survival, angiogenesis, and metastasis in epithelial tumors, suggesting a role for Postn in tumor progression [13,15,17,18]. Recent clinical evidence has also revealed that Postn is overexpressed in breast cancers [19] and involved in the progression of mammary tumors to invasive and metastatic cancers. More importantly, acquired expression of Postn by breast cancers is associated with increased angiogenesis and metastasis [20,21]. Recently, Postn has been found to be critical for the establishment of tumor cell niche and the reactivation of dormant tumor cells [22,23]. Interestingly, Postn appears to play a role in regulating the availability of Wnt factors to tumor-initiating cells [22].

The androgen receptor (AR) plays an important role in hormone-dependent cancers [24]. In most cases, AR usually triggers its action by binding to testosterone and activating gene expression following nuclear translocation. Interestingly, the AR has recently received much attention as a novel therapeutic target in breast cancer [24-26]. AR expression and phosphorylation has been observed in a number of breast cancers [25,27]. However, confusing results have emerged from the analyses of the various breast cancer subtypes. Surprisingly, high levels of AR activity have been associated with better outcomes in estrogen receptor (ER)-positive cancers but with poor prognosis in ER-negative and HER2+ cancers [26,28-30]. These findings suggest a role for AR activation in a proportion of HER2+ cancers.

In polyoma middle T (PyMT)-induced mammary tumors, Postn was found to be dispensable for primary tumor initiation and growth [22] but required for lung metastasis. As the various murine models of breast cancer display differences in latency and progression, we tested the role of Postn in MMTV-Neu (NDL2-5) mice [31], expressing an activated Neu/ErbB2/HER2 from the MMTV promoter. As for MMTV-PyMT mice, our results show that Postn deletion does not affect mammary gland development or tumor initiation in MMTV-Neu mice. However, tumors from Postn-null mice exhibit apocrine-like features and express low levels of active Notch1 but high levels of AR and prolactin-induced protein (PIP). Mammary tumor cells derived from wild-type MMTV-PyMT mice (Met1) failed to grow in Postn-null mice when injected subcutaneously. However, re-expression of active Notch1 or PIP in Met1 tumor cells rescued the growth deficit in a Postn-null environment, suggesting that the loss of Postn selects for an AR-dependent pathway. Overall our results show that Postn deletion results in decreased Notch1 levels and a switch to a molecular apocrine subtype in a Her2-positive context.

## Materials and methods

### Animal analysis and genotyping

MMTV-NeuNDL were as described previously [32]. Postn(−/−) mice in a C57Bl/6 background were backcrossed into FVB/N mice. The FVB/N contribution was evaluated after two generations using Marker-assisted Accelerated Backcrossing (MAX-BAX™; Charles River Laboratories, Wilmington, MA, USA). Heterozygote Postn(+/−) mice from the 3^rd^ backcross (95% FVB/N) were used for subsequent breeding to MMTV-NeuNDL males to obtained the desired genotypes. All animals were genotyped by polymerase chain reaction (PCR) analysis. Mouse DNA was extracted from mouse ear clippings and purified using the DNeasy™ blood and tissue kit according to the manufacturer’s protocol (Qiagen, Venlo, Netherlands). For Postn genotyping, three primers were used. Primer 1: 5′ – AGTGTGCAGATGTTTGCTTG – 3′, primer 2: 5′ – ACGAAATACAGTTTGGTAATCC – 3′, and primer 3: 5′ – CAGCGCATCGCCTTCTATCG – 3′. Genotyping of MMTV-NeuNDL mice was performed using the following primer pair: 5′ – GTTTCCTGCAGCAGCCTACGC – 3′ and 5′ – TTCCGGAACCCACATCAGGCC – 3′.

For weight determination, Postn^+/+^, Postn^+/−^, and Postn^−/−^ mice were weighed with a scale twice a week for 9 weeks to establish a growth curve. Weights were averaged to establish the data points. NeuNDL Postn^+/+^, NeuNDL Postn^+/−^, and NeuNDL Postn^−/−^ mice were palpated once a week, every week starting at 4 months of age. A total tumor burden of 1.7 cm^3^ was considered as the end point as per the University of Ottawa guidelines. Animal studies were approved by the University of Ottawa animal ethics board (NSI-73). Care and use of experimental mice followed the guidelines established by the Canadian Council on Animal Care.

### Expression vector and tissue culture

The MMTV-PyMT-derived Met-1 cells [33] were provided by A. Borowsky and maintained in Dulbecco’s modified Eagle’s medium (DMEM)/10% fetal calf serum (FCS) at 37°C in 5% CO_2_. The murine Postn cDNA was a kind gift from A. Kudo, (Tokyo; [13]). The Notch-ΔNLS expression vector was provided by P. Jolicoeur (Montreal, IRCM) and the NICD cDNA was obtained from Addgene (Cambridge, MA, USA). Dominant-negative MAML1 was kindly provided by J. Aster (Boston, Harvard). The PIP cDNA was PCR amplified (5′-CCCTCGAGATGCAGGGTCTCTCATTCAC- 3′ and 5′- CCGAATTCTTAATTCATTCGCACAG TATTA - 3′) and all expression vectors were generated in the pLPCX retroviral backbone. Retrovirus using individual vectors was generated using the Plat-E retroviral packaging cell line and used to transduce Met1 cells as described previously [34,35] and puromycin-resistant pools (1ug/ml; Calbiochem, San Diego, CA, USA) were expanded and assessed for expression by Western blot (Postn and NICD) or Q-PCR (dnMAML1, ΔNLS and PIP). For heterotopic transplants, 10^6^ cells were injected subcutaneously into the flanks of 10- to 12-week-old FVB/N female mice (n = 5). Tumor volumes were monitored weekly for 28 days using calipers.

### Western blot analysis and antibodies

Mammary tumors or mammary glands were crushed in liquid nitrogen with a mortar and pestle and then transferred to an Eppendorf tube and lysed in RIPA buffer (50 mM Tris-HCl (pH 7.5), 150 mM NaCl, 1.0% Triton-X, 1.0% Nonidet P-40, 0.5% sodium dideoxycholate, 0.1% sodium dodecyl sulfate (SDS), 2 mM EDTA), containing 10 mM NaF, 1 mM DTT, 10 μg/ml leupeptin, 10 μg/ml aprotinin, 0.1 mM benzamidine, 10 mM β-glycerol phosphate, 1 mM PMSF, 0.25 mM Na_3_VO_4_, and 10 μg/ml pepstatin. Protein concentrations were measured using the Bio-Rad protein assay dye reagent (Bio-Rad Laboratories, Hercules, CA, USA). Samples containing 40 μg of total protein were resolved by SDS-PAGE and transferred to polyvinylidene fluoride (PVDF) membrane (PerkinElmer, Waltham, MA, USA). Membranes were then probed with various antibodies as previously described [36]. The primary antibodies were detected using conjugated horseradish peroxidase (HRP)-labeled secondary antibodies and detected using Western Lightning™ Plus-ECL enhanced chemiluminescence (PerkinElmer). Reactive bands were visualized by exposure to X-ray film. Protein expression levels were quantified using densitometric analysis using ImageJ and normalized to tubulin expression. Statistical analysis was performed using a two-tailed unpaired Student’s *t* test.

The following antibodies were purchased and used according to the manufacturer’s protocols: Postn (R&D Systems, Minneapolis, MN, USA), pAkt-S473, Akt, NICD, Notch1 (Cell Signaling, Danvers, MA, USA), cyclin D1, (Santa Cruz Technology, Dallas, TX, USA), pY397FAK, focal adhesion kinase (FAK) (BD Biosciences, San Jose, CA, USA), androgen receptor, CD31 (Abcam, Cambridge, UK), ErbB2 (Calbiochem, clone ab-3), Ki67, non-phospho beta catenin (EMD Millipore, Billerica, MA, USA), tubulin (Sigma-Aldrich, St Louis, MO, USA), γ-secretase inhibitor, (In solution inhibitor X; Calbiochem).

### Immunohistochemical analysis and mammary whole mount

For mammary gland whole mounts, the 4^th^ inguinal mammary gland was excised processed for hematoxylin staining as described [37]. For immunohistochemistry, the mammary glands and mammary tumors were excised and fixed in 10% formalin buffer overnight at room temperature. The samples were then paraffin-embedded and sectioned onto microscope slides. The mammary gland and mammary tumor sections were deparafinized and heat-mediated antigen retrieval was performed using 10 mM citrate buffer followed by incubation with a primary antibody in 1.5% normal goat serum at 4°C overnight in a humidified chamber. The sections were then washed and incubated with biotin- or HRP-conjugated secondary antibody diluted in 1.5% normal goat-blocking serum at room temperature for 30 minutes. Color development was achieved using DAB followed by hematoxylin counterstain. Human tissue microarrays (TMA; BR962) were purchased from US Biomax, Inc (Rockville, MD, USA) and stained for Postn.

### Microarray, quantitative PCR analysis and luciferase assays

RNA was prepared according to the manufacturer’s protocol by directly lysing frozen tumors and cultured monolayers into Trizol (Invitrogen, Carlsbad, CA, USA). The RNA was then treated with DNaseI (Qiagen) to remove any contaminating genomic DNA. For Affymetrix microarrays, 5 μg of total RNA from two independent tumors for both wild-type and Postn(−/−) were subjected to hybridization to the Mouse Gene 1.0 ST array v.1 (Affymetrix, Santa Clara, CA, USA). The raw output data analysis was performed using the online tool WEBARRAY - Online Microarray Data Analysis - in ‘Linear model statistical analysis’ mode. The data was normalized using the RMA algorithm. For cDNA synthesis, 1 μg of the resulting RNA was used in a 20 μl RT-PCR reaction using SuperscriptIII reverse transcriptase (Invitrogen) following the manufacturer’s protocol. The resulting cDNA was used in quantitative real-time polymerase chain reaction (qRT-PCR) using SYBR Green chemistry (Bio-Rad). Q-PCR was performed in a 96-well format using an Applied Biosystems 7500 real-time PCR system (Applied Biosystems, Waltham, MD, USA). The primers used are listed in Table S1 in Additional file 1.

Wnt activity measurements in Met1 pools stably expressing Postn or NICD cDNAs were performed by cotransfection with the Top flash/Renilla or Fop flash/Renilla plasmid system and luciferase assays were performed using the Dual-Glo™ Luciferase Assay System (Promega, Madison, WI, USA) according to the manufacturer’s instructions.

### Statistical analysis

Animal survival was subjected to Kaplan-Meier analysis and significance was evaluated using the log rank test. For tumor volume measurements, the average tumor volume from five mice was plotted over time and analyzed using a two-tailed unpaired Student’s *t* test. Q-PCR gene expression data were normalized to GAPDH levels and calculated using the comparative Ct (ddCt) method and the significance in the difference of the means between the groups was calculated using a two-tailed unpaired Student’s *t* test. The proportion of nuclear AR was calculated from at least 1,000 nuclei from three independent tumors, compared using Student’s *t* test and shown as the average and standard error of the mean (S.E.M). Results were considered statistically significant at *P* <0.05.

## Results

### Postn is not required for mammary gland development

Previous reports have shown that Postn-null mice are viable but display impaired periodontal ligament development [14,38,39]. Therefore, prior to the introduction of the Postn-null allele into MMTV-NeuNDL mice, we assessed Postn expression and whether Postn ablation was compatible with normal mammary gland development. Mice carrying a LacZ knock-in [14] into the Postn locus were bred into an FVB/N background and assessed for Postn expression and mammary gland development in virgin and pregnant females. Postn protein isoforms [40] are readily detectable in the mammary glands of 8-week-old virgin wild-type females but absent from Postn-null glands (Figure S1 in Additional file 2). Mammary gland whole mount analyses (Figure S1 in Additional file 2) revealed normal branching and ductal outgrowth in Postn(−/−) nulliparous females, indistinguishable from that of control mice. Similarly, 12-week-old pregnant (14.5dpc) Postn-deficient females displayed extensive outgrowth and arborization that was similar to wild-type or Postn(+/−) littermates (Figure S1 in Additional file 2). Histological analysis by hematoxylin and eosin (H&E) staining of nulliparous mammary gland showed no defects in the ductal epithelial and stromal layers (Figure S1L-O in Additional file 2). Supporting this, we observed that FVB/N Postn^−/−^ female mice were able to lactate and produce viable litters through multiple rounds of pregnancy (data not shown). However, in contrast to what has been observed in a C57/Bl6 background [14], the viable Postn-null pups displayed a mild growth retardation phenotype that became negligible by 9 weeks of age (data not shown). Together, these results suggest that Postn is not required for normal mammary gland development.

Postn has been shown to be expressed in the stromal and epithelial compartment of the embryonic heart [41]. Similarly, Postn expression was detected in both the stroma and tumor cells of human breast cancer samples [19-21]. Therefore, the cell type-specific expression of Postn in the mouse mammary gland was investigated using immunohistochemical analysis of paraffin-embedded sections. Examination of mammary tissue derived from Postn^+/+^ and Postn^+/−^ mice revealed that Postn is expressed in the stromal cells lining the ducts and lobules, but not in the mammary epithelial cells (Figure S1 in Additional file 2). Postn reactivity was also observed in the intercellular space between the adipocytes.

### Loss of Postn does not affect survival in NeuNDL mice

**Figure 1:**
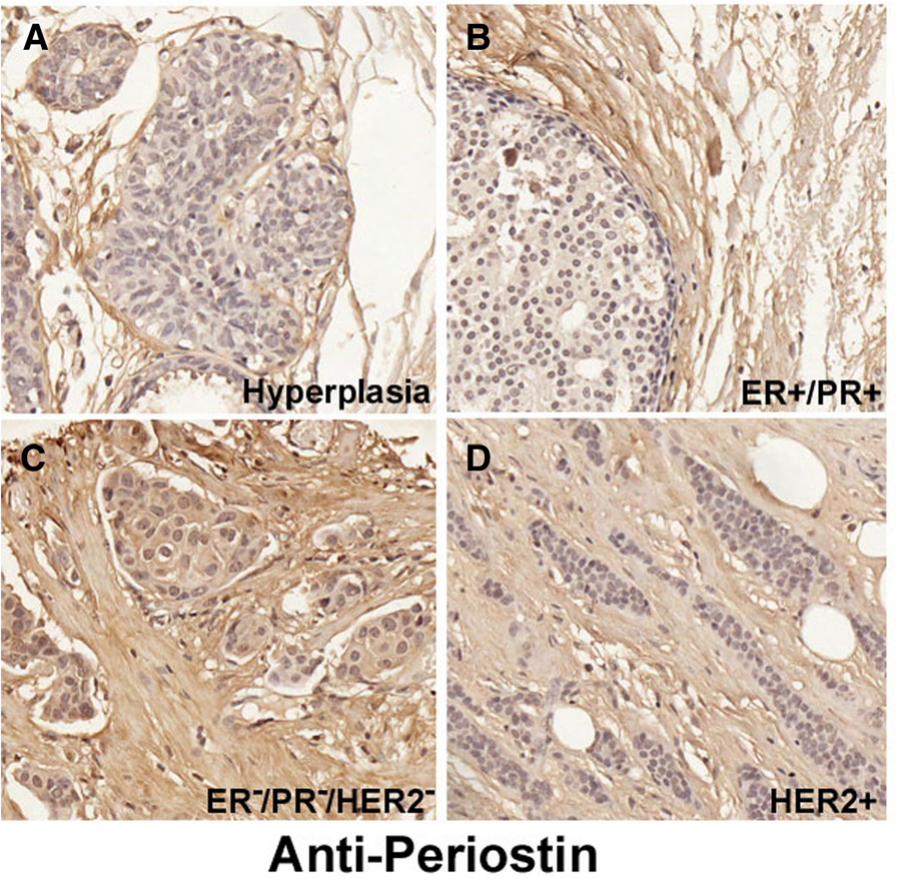
**Postn expression in human breast cancers.** Anti-Postn immunohistochemistry showing Postn expression in the stroma of human mammary glands bearing hyperplastic lesions **(A)**. However, some mammary tumors were found to acquire Postn expression independently of receptor status. Representatives of Postn-negative **(A-B)** and positive **(C-D)** are shown. The expression data are summarized in Table 1.

Postn has been shown to play multiple roles in tumor progression through activation of pathways involved in invasion, cellular survival, angiogenesis, and metastasis [15,42]. Furthermore, Postn expression levels were found to be increased in a high proportion of breast cancers [19-21]. Supporting this, our analysis of human tissue microarray (Figure 1) revealed that about 46% (16/35) of human breast cancers acquire Postn expression irrespective of receptor status (Table 1).

**Table 1:** **Biomax US BR962 breast cancer TMA was stained with an anti-Postn antibody (see Figure** 1 **) and the results were scored as a percentage of the total cores on the TMA**

**BR962**	Postn + ve*	**AR + ve**
**35 Tumors**	16/35 (46%)	3/16 (9%)^#^
**13 Non-malignant**	0/13 (0%)	10/13 (77%)^§^

^*^Indicates the percentage of cores that displayed Postn expression by the cancer epithelia (see Figure 1G). ^#^The AR expression scores of the three cores were reported to range between 5 and 30% positivity by the manufacturer. ^§^All but three cores were reported to range between 1 and 50% positivity by the manufacturer. Expression levels were reported as variable. TMA, tissue microarray; Postn, periostin; AR, androgen receptor.

**Figure 2:**
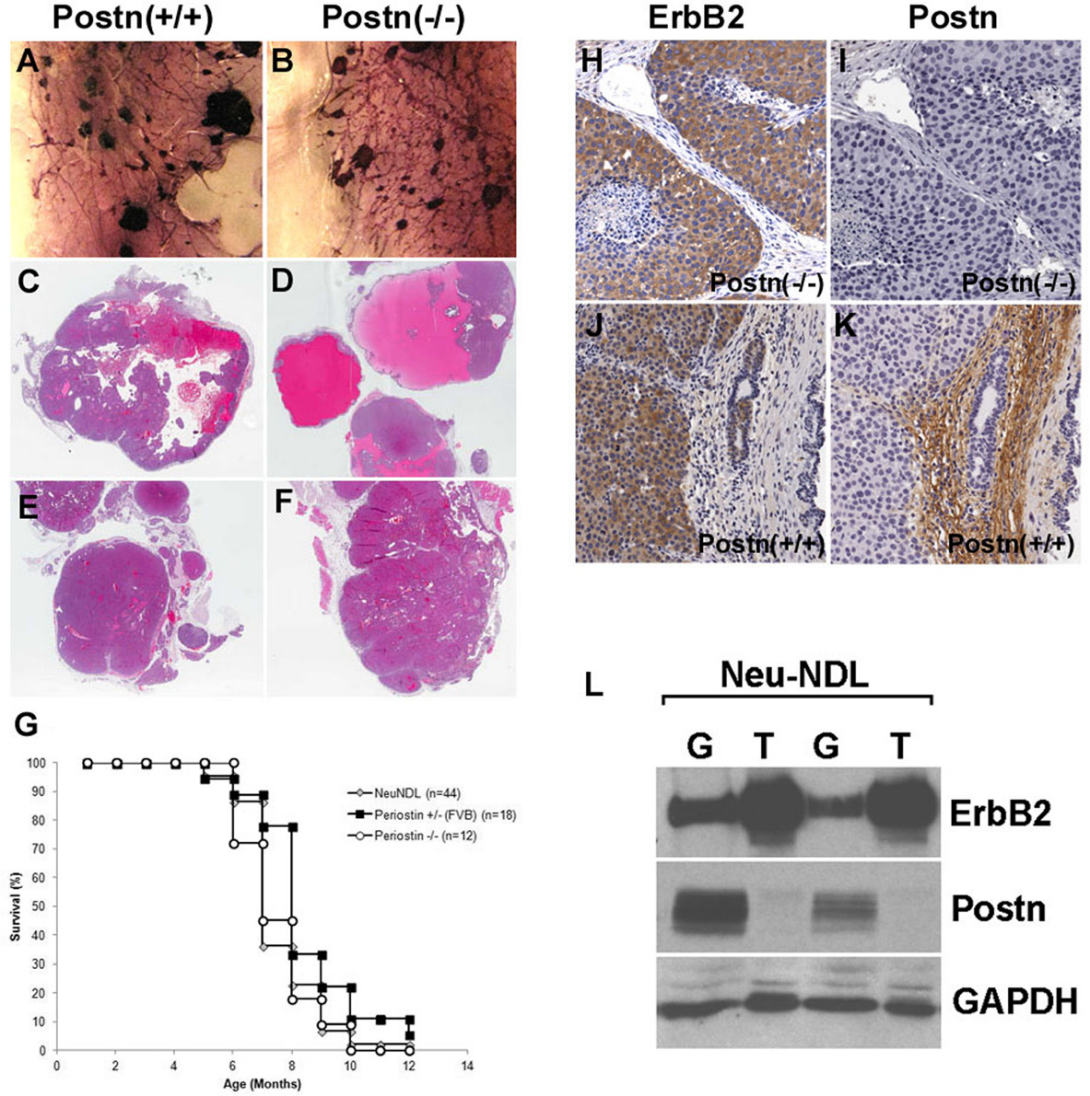
**Loss of Postn does not affect tumor growth in MMTV-Neu mice. (A and B)** Representative mammary gland whole mounts from 4 month old virgin females showing multiple tumor foci in both wildtype and Postn(-/-) mice. **(C-F)** H & E staining of representative tumors collected at end point from wildtype **(C and E)** and Postn-null **(D and F)** mice. Both cystic **(C and D)** and solid tumors **(E and F)** were observed. **(G)** Kaplan-Meier survival analysis of tumor-bearing mice. Log rank testing showed no significant delay in tumor progression for Postn(-/-) mice when compared to heterozygotes or wildtype mice. All three genotypes reached end point with similar kinetics. **(H-K)** Paraffin-embedded sections were stained for ErbB2 **(H and J)** or Postn **(I and K)**. Tumors arising in Postn-null females retained Neu expression. In Neu-induced tumors, Postn expression was restricted to the stromal compartment and was never found in the tumor cells. **(L)** Western blot analysis of mammary tumors (T) and whole gland **(G)** lysates. Supporting the IHC data, Postn was found to be expressed at low levels in tumors containing a small amount of stromal fibroblasts.

Therefore, to investigate whether Postn expression is required for mammary tumorigenesis in an ErbB2-positive model, the in-frame Neu deletion transgene under the MMTV promoter (MMTV-NeuNDL2-5) [31] was introduced into mice lacking Postn. Postn wild-type, heterozygotes and null NeuNDL virgin females were monitored for tumor development. In all Postn genotypes, hyperplastic lesions or palpable tumors could be detected as early as 16 weeks of age with no differences in the number of observed lesions (Figure 2A,B and Figure S2 in Additional file 3). Similarly, no differences were observed in tumor growth as all the Postn genotypes reached end point with similar kinetics (Figure 2G). Further analysis of NeuNDL-Postn^+/+^ and Postn^−/−^ tumors revealed a mixture of intracystic or encapsulated intraductal papillary carcinomas (Figure 2C-F) that retained Neu expression (Figure 2H,J). In addition, no changes were observed in CD31-positive tumor blood vessels or in the Ki-67 proliferative index (Figure S3 in Additional file 4). These findings suggest that Postn ablation does not prevent or delay primary tumor initiation or growth. Similar findings have been reported in the MMTV-PyMT mouse model [22].

In human breast tumor tissues, Postn was reported to be highly expressed in the stromal cells immediately surrounding the tumor and in late-stage mammary tumors [19,21]. Supporting this, immunohistochemical analysis of Postn in NeuNDL Postn^+/+^ and Postn^+/−^ tumors shows expression in the stromal compartment around the epithelial mammary tumors. Interestingly, Postn was undetectable in the mammary tumor cells (Figure 2K). Similarly, Western blot analysis of mammary glands and tumors shows higher levels of Postn in whole mammary gland containing a higher proportion of mammary fibroblasts (Figure 2L). Together, these results suggest that Postn expression is restricted to the stromal compartment of NDL tumors and is not upregulated in the tumor epithelium in MMTV-Neu-NDL mice.

**Figure 3:**
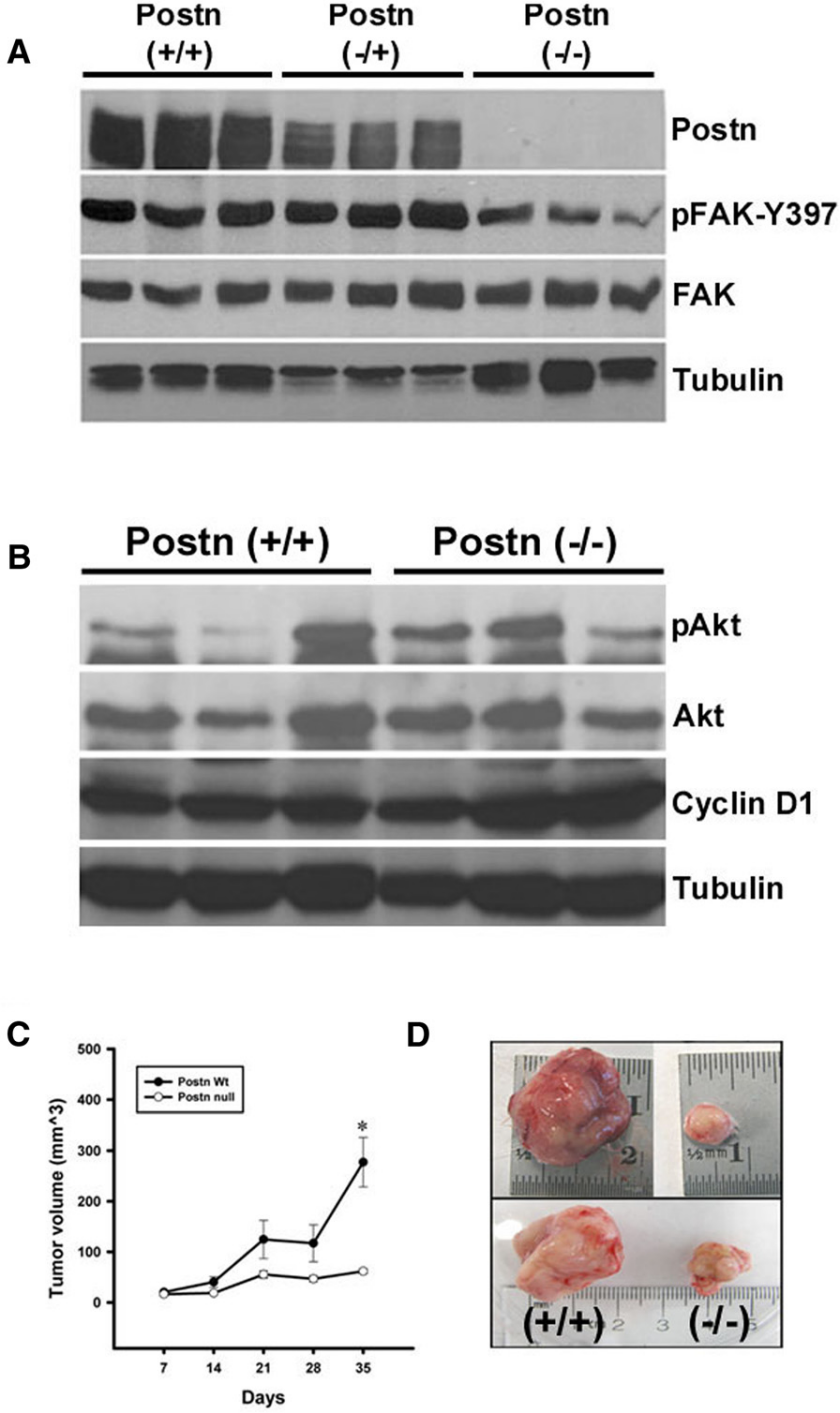
**Loss of Postn alters FAK signaling and delays tumor growth at heterotopic sites. (A)** Total cell lysates from 3 independent tumors were surveyed for FAK activation. Lower levels of FAK activation (pFAK-Y397) were observed in Postn-null tumors. **(B)** Western blot analysis for activated Akt (pAkt; S473) and Cyclin D1 showed no differences in Postn(−/−) tumors when compared to wild-type. **(C)** Tumor volume measurements following the subcutaneous injection of 10^6^ Met-1 cells into the flank of FVB/N wildtype or Postn-null female mice (n=5). No appreciable growth was observed in Postn-null mice suggesting that Postn is required for tumor progression at heterotopic sites. ^*^ *P <0.05* **(D)** Representative tumors excised at 35 days following the injections of Met-1 cells as described in **(C)**.

To further investigate the effect of Postn deletion in mammary tumorigenesis, we surveyed a number of markers activated downstream of Postn treatment. Postn stimulation has been previously shown to activate FAK and Akt through β3 or β4 integrin binding [16,43,44]. Western blot analysis showed an overall 2-fold decrease in the levels of phospho-FAK-Y397 protein kinase (Figure 3A). However, β3 integrin levels remained unchanged in Postn-null tumors (not shown). Interestingly, the levels of phospho-Akt-S473 were not affected, suggesting that the loss of Postn can preferentially affect specific pathways in mammary tumors. Similarly, ErbB3 levels were unaffected (not shown). Supporting our Ki-67 observations, cyclin D1 levels also remained unchanged in tumors derived from Postn-null animals (Figure 3B).

### Loss of Postn results in reduced Notch1 activity

Although Postn has been shown to be overexpressed in a high proportion of human tumors and to play a role in tumor cell growth *in vitro* [15,19-21,42], Postn deletion has no effect on tumor development in ErbB2- (Figure 2) or PyMT-expressing mice [22]. However, previous data have shown that Postn plays a critical role in the establishment of tumor cell niche at secondary sites [22]. Supporting this, administration of Postn neutralizing antibodies in xenograft models of breast and ovarian cancers also suppressed invasion and metastasis [45,46].

Therefore, in light of these observations we tested whether mammary tumor cells injected at heterotopic sites could form tumors in a Postn-null environment. As skin tissue is a Postn-rich environment [47,48] we tested the requirement for Postn in tumor growth using subcutaneous injections. As PyMT also activates ErbB2 downstream signaling during tumorigenesis [49], we used MMTV-PyMT-derived Met-1 cells [33] in an isograft model.

Whereas large tumors were observed in Postn wild-type females, little or no growth was observed following the subcutaneous injection of Met-1 cells into Postn-deficient animals. Tumor volume measurements showed that Met-1 cells in a wild-type environment grew to about 10 times the size of the tumors collected following injections into Postn-null mice (Figure 3C,D). In the Postn-null background, no significant increase in tumor growth was observed up to 45 days whereas all wild-type mice had to be euthanized beyond 5 weeks due to very large tumors (data not shown). Interestingly, exogenous expression of Postn in Met-1 cells did not rescue their growth defect in a Postn-null environment. As those animals never expressed Postn protein, this is likely due to immune rejection of Postn-expressing cells in null mice. These data strongly suggest that Postn is required for sustaining tumor growth at heterotopic sites.

Postn has been shown to interact with Wnt and to potentiate Wnt signaling to enhance tumor cell seeding [22]. Interestingly, Western blot analysis for β-catenin (or active β-catenin; not shown) levels, nuclear localization or activity showed no difference in tumors derived from Postn-null mice (Figure S4 in Additional file 5), suggesting that the loss of Postn does not affect canonical Wnt signaling in ErbB2-driven tumors. Furthermore, this suggests that the impaired tumor growth observed in Postn(−/−) mice is unlikely due to deficient Wnt signaling.

Previous studies have shown that the lack of Postn during embryonic development results in aortic valve defects [50]. These anomalies are due to increased expression of the Notch1 antagonist Dlk1 and suppression of Notch signaling. Similarly, Postn association with Notch1 has been found to maintain Notch1 levels under stress conditions [51]. As Notch also plays an important role in mammary carcinoma and stem cell function [52-55], we investigated whether Postn-deficient tumors also displayed alterations in the Notch pathway. As Dlk1 mRNA levels were unaffected (not shown), we directly assessed the levels of active Notch and its downstream target Hey1 in mammary tumors derived from wild-type and Postn-deficient animals.

**Figure 4:**
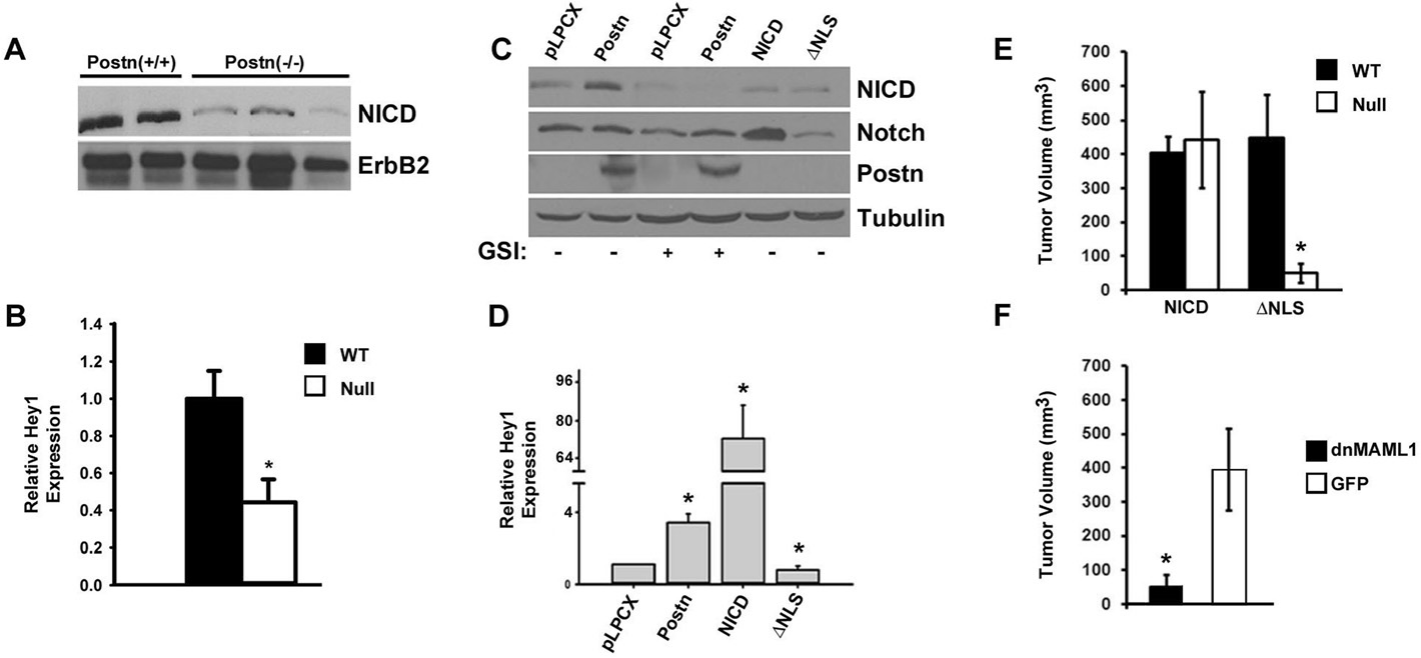
**Active Notch expression can bypass the Postn requirement for heterotopic growth of Met-1 cells. (A)** Western blot analysis of tumor lysates showing a marked reduction of active Notch levels in Postn-null tumors. This is accompanied by a 2-fold reduction in the levels of Hey1 gene expression as measured by Q-PCR **(B**; ^*^ *P* <0.01**)**. The data shown are an average of 4 independent tumors performed in triplicate. **(C)** Western blot analysis of Postn- or NICD-expressing Met-1 cells. Cells expressing Postn showed a marked increase in endogenous NICD levels when compared to vector control (pLPCX). The increase in endogenous NICD was blocked by the addition of a γ-secretase inhibitor (GSI). Overexpression of exogenous NICD was only detectable with a total Notch antibody as the anti-NICD epitope has been deleted in the construct. Similarly, expression of the NLS mutant can only be detected by Q-PCR as it lacks both epitopes recognized by the commercial anti-NICD and anti-Notch antibodies. **(D)** Quantitation of Hey1 mRNA levels showed a 4- and 70-fold increase in Met-1 cultures overexpressing Postn or NICD, respectively (^*^ *P* <0.01). No increase was observed in ΔNLS-expressing cells. **(E)** Tumor volume measurements at 28 days post-injection of Met-1 cells overexpressing NICD or ΔNLS in wildtype or Postn-null mice. Cells over-expressing NICD could bypass the Postn requirement for subcutaneous growth in FVB/N females. Little or no growth was observed for ΔNLS-expressing cells (^*^ *P* <0.01). **(F)** Expression of dnMAML1 in Met-1 cells impairs their growth in wildtype mice when compared to a GFP-expressing control (^*^ *P* <0.01).

Western blot analysis revealed a 3-fold decrease in the levels of the transcriptionally active Notch intracellular domain fragment (NICD; reviewed in [52]) (Figure 4A). This was accompanied by an approximately 50% downregulation in Hey1 mRNA levels, a NICD target gene (Figure 4B). However, Hes1 levels remained unchanged (not shown), supporting the notion that Notch-mediated activation of Hes1 transcription is context and cell type-dependent [56]. Together our results suggest that the loss of Postn is accompanied by reduced Notch1 activity and a failure to support tumor growth at secondary sites.

### Postn drives tumor growth by activating Notch1

Our data suggest that high Notch activity is required for tumor growth at heterotopic sites. Therefore, we tested whether increased Notch activity was sufficient to bypass the Postn requirement in our heterotopic model. Met-1 cells stably expressing NICD were generated and compared to Met-1 expressing Postn or a nuclear localization signal mutant NICD construct (ΔNLS; Figure S5 in Additional file 6). In cell culture, overexpression of Postn in Met-1 cells resulted in a γ-secretase-dependent upregulation of NICD protein levels (Figure 4C), suggesting that Postn signaling in mammary tumor cells can contribute to the regulation of active Notch levels. The upregulation of NICD in Postn-expressing cells was accompanied by a 4-fold increase in Hey1 mRNA levels (Figure 4D). Met-1 cells expressing NICD upregulated Hey1 mRNA by more than 50-fold. In contrast, no induction was observed in control cells expressing the NLS mutant form of NICD. When injected subcutaneously into Postn-deficient FVB/N female mice, Met-1 cells expressing NICD grew as well as in wild-type mice, suggesting that NICD re-expression is sufficient to bypass the Postn requirement observed for control cells (Figure 4E). Interestingly, in wild-type mice, NICD overexpression did not enhance tumor growth beyond what was observed for control cells, suggesting that the levels of downstream NICD effectors might be limiting. Expression of the inactive NICD could not rescue the Met-1 growth defect in Postn-null mice, suggesting that Notch activity is required.

Interestingly, Postn or NICD expression in Met-1 cells did not enhance the activity of a β-catenin reporter gene (Figure S4 in Additional file 5), suggesting that Notch1 does not bypass the Postn requirement by indirectly stimulating the Wnt system. Although Notch1 has been previously shown to play a role in the expansion of premalignant and tumor-initiating cells in NICD-induced mammary tumors [55,57], we did not find any differences in the primary or secondary tumorsphere forming ability of primary tumors derived from Postn-null mice (Figure S6 in Additional file 7), suggesting that the loss of Postn has no effect on the primary tumor stem cell compartment.

To test the requirement for the Notch pathway in a wild-type environment, a dominant-negative form of the Notch co-activator Mastermind-like 1(dnMAML1; [58]) was overexpressed in Met-1 cells. Expression of dnMAML1 in Met-1 cells resulted in Hey1 downregulation and poor tumor growth upon subcutaneous injection (Figure 4F). Furthermore, expression of dnMAML1 resulted in a 50% decrease in tumor take rate when compared to GFP-expressing control cells (GFP: 5/6 vs dnMAML1: 8/16). Together, these data suggest that Postn is necessary to maintain a threshold level of active Notch, required for tumor establishment and growth at heterotopic sites.

### Loss of Postn results in an androgen receptor-positive apocrine-like phenotype

**Figure 5:**
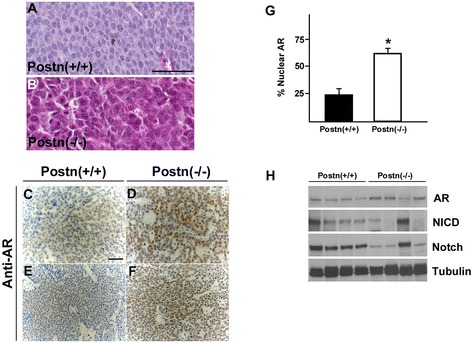
**Loss of Postn leads to increased AR levels and activity. (A)** H&E staining of primary tumors derived from wildtype **(A)** and Postn-null mic **(B)**. Postn-deficient tumors displayed an apocrine-like morphology with granular eosinophilic cytoplasm and prominent nucleoli. **(C-F)** Immunohistochemical analysis of independent tumors from Postn-null mice shows an increase in AR levels and nuclear localization **(G)**. At least 1000 nuclei from 3 independent tumors were assessed for AR nuclear translocation. The average and S.E.M. is shown. ^*^ *P* <0.05. **(H)** Western blot analysis of Postn-null tumors reveals that Notch downregulation results in AR upregulation. Note one tumor sample where low AR levels can be correlated to high levels of Notch.

Although loss of Postn did not affect overall survival and tumor development, our results show that it impaired tumor growth at heterotopic sites. In addition, careful histological examination of these tumors revealed a molecular apocrine-like phenotype, characterized by abundant granular eosinophilic cytoplasm, vesicular nuclei with prominent nucleoli (Figure 5A,B). The molecular apocrine phenotype is a characteristic of androgen receptor (AR)-positive breast cancers [59,60]. Therefore, we assessed AR protein levels in wild-type and Postn-null NeuNDL tumors by Western blot analysis. Although AR mRNA levels were unchanged (not shown), AR protein levels were found to be upregulated 2-fold in the majority of tumors derived from Postn-null mice (Figure 5H). Furthermore, immunohistochemical analysis for AR expression revealed a significant increase in nuclear localization of AR in tumors from Postn-null animals, suggesting an increase in AR activity (Figure 5C-G). The increase in AR protein levels in Postn-null tumors suggests a posttranscriptional upregulation or stabilization of AR protein levels [61,62]. Interestingly, we have identified one tumor sample where AR levels were unchanged which could be correlated with wild-type levels of active and total Notch. Together these findings demonstrate that the loss of Postn results in Notch downregulation which is accompanied by AR upregulation in the primary tumors. Furthermore, this suggests that the loss of Postn in ErbB2-expressing tumors confers an AR+ apocrine-like phenotype.

To gain further insights into the molecular mechanisms regulating tumor progression in a Postn-null environment, we compared the gene expression profiles of Postn(−/−) and Postn(+/+) tumors using Affymetrix microarrays. Table S2 in Additional file 1 shows the genes that were up- or downregulated by at least 10-fold in Postn-null tumors. Supporting the histological findings, numerous genes that have been previously reported as AR target genes, such as the androgen-binding proteins [63] and PIP [64] were upregulated in Postn-null tumors. These observations suggest that the activation of critical genes in the absence of Postn allows primary tumor growth in the absence of high Notch activity.

**Figure 6:**
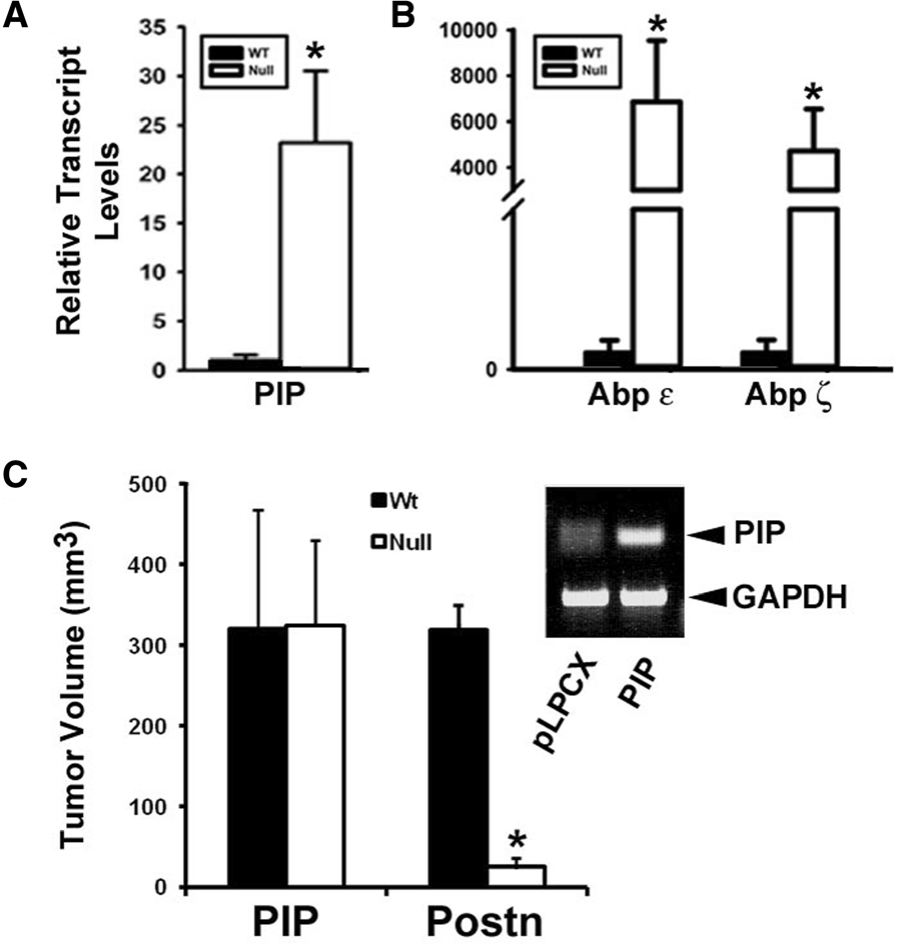
**The AR target gene PIP rescues tumor growth in Postn-null mice.** Q-PCR expression analysis shows that the AR target genes prolactin-induced protein (PIP; ^*^ *P* <0.05) **(A)** and androgen-binding proteins ε and ζ **(B)** are markedly induced in Postn-null tumors (^*^ *P* <0.005). **(C)** Tumor volume measurements at 28 days post-injection of PIP- or Postn-expressing Met-1 cells. Expression of PIP in Met1-cells rescues their subcutaneous growth defect in FVB/N Postn-null females (^*^ *P* <0.01). Postn-expressing cells could not rescue the growth deficit in Postn(-/-) mice. Inset: Ethidium bromide-stained PCR reaction showing expression of exogenous PIP in Met-1 cells.

In human tumors, PIP (PIP/GCDFP15) expression was shown to be associated with AR+ molecular apocrine-like tumors [65-67]. To confirm the array data, Q-PCR was performed on tumor RNA to validate PIP overexpression in Postn-null tumors. In addition, we assessed the mRNA levels of androgen-binding proteins (Abp), previously shown to be upregulated by testosterone in cultured cells [63]. Quantitative PCR analysis revealed that both PIP and Abp (ε and ζ) were significantly upregulated in Postn-deficient tumors (Figure 6). Whereas PIP was increased by about 20-fold (Figure 6A), Abp ε and ζ were upregulated by more than 1,000-fold (Figure 6B), suggesting transcriptional activation of AR target genes in tumors derived from Postn-deficient animals.

Interestingly, in addition to being an AR-regulated gene [64], PIP/GCDFP-15 has been demonstrated to be expressed in a large proportion of human breast cancers and to enhance the growth and invasion of breast cancer cell lines [68-71]. In addition, PIP was found to activate AR activity, which in turn, upregulates PIP transcription in feed-forward loop [70]. As PIP is an AR target gene that is highly induced in tumors from Postn-null mice, we tested the possibility that PIP expression in wild-type Met-1 cells could compensate for the absence of Postn *in vivo*. The murine PIP cDNA was cloned and stably expressed in Met-1 cells and pools were injected subcutaneously in Postn-null female mice. Similar to NICD-expressing cells, at the 4-week end point, Met-1 cells overexpressing PIP formed large tumors in Postn-null mice that were comparable in size to those observed in wild-type animals (Figure 6C). Control puromycin-resistant cells grew very poorly in the Postn-deficient environment. These results suggest that PIP is a major downstream effector of AR activation and that its expression in Met-1 cells is sufficient to overcome the Postn deficiency *in vivo*. However, mechanistically, it is likely that the rescue by NICD and PIP proceeds through distinct pathways.

Further, AR levels did not change in control and NICD expressing subQ tumors in Postn-null mice as compared to subQ tumors grown into wild-type mice (Figure S5B in Additional file 6), suggesting that the NICD growth rescue in null mice does not result in an apocrine phenotype. Together, our results show that the loss of Postn leads to Notch downregulation and selects for AR-positive molecular apocrine-like tumors.

## Discussion

**Figure 7:**
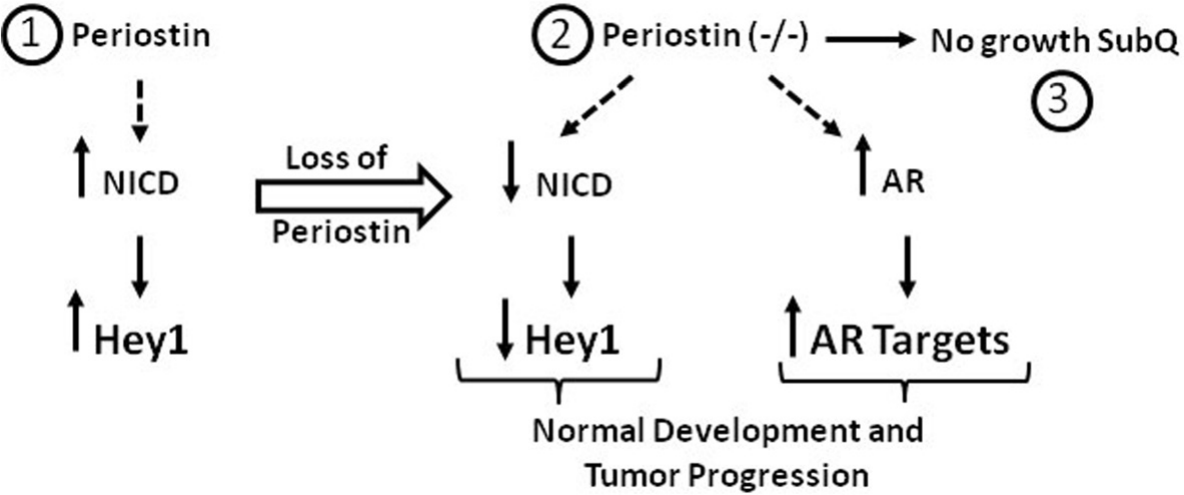
**Loss of periostin activates an AR response.** Through an unknown mechanism, the presence of Postn in the microenvironment stimulates or maintains Notch processing and NICD activity (1). Loss of periostin (2) results in NICD downregulation and AR upregulation by an undefined mechanism (dotted lines). This is accompanied by Hey1 downregulation and the activation of AR target genes without any effect on development. Activation of the AR pathway or Notch signaling is sufficient to drive normal mammary tumor progression. However, in a Postn-deficient environment tumor growth at heterotopic sites is impaired (3).

HER2/ErbB2/Neu is overexpressed in about 30% of human breast cancers and is associated with poor prognosis (reviewed in [5]). As high levels of Postn have been found to be associated with breast cancer progression [19-21], we have derived Postn-deficient mice in the MMTV-Neu background. Supporting previous findings [22], our data show that the loss Postn does not impair mammary gland development, suggesting that it can proceed through Postn-independent mechanisms. In addition, Postn deletion did not affect the onset or progression of mammary tumors in FVB/N mice. Surprisingly, wild-type Met-1 cells grew very poorly in a Postn-deficient environment. This growth defect was rescued by re-expressing active Notch1, suggesting that Postn is required to maintain Notch1 activity (see Figure 7). Supporting this, expression of a dominant negative Notch co-activator, MAML1, suppressed heterotopic tumor growth in a wild-type environment, suggesting that the Notch pathway plays a critical role in this process. Although Notch activity appears to be regulated by Dlk1 levels in cardiac tissues from Postn-null mice, we did not observe any changes in Dlk1 or Notch1 mRNA levels. Postn has been demonstrated recently to interact with Notch and to induce its processing under hypoxic conditions [51]. Deletion of Postn in periodontal ligaments leads to a decrease in Notch processing and increased cell death under stress conditions. One possibility is that in the tumor microenvironment, a similar interaction is required to maintain Notch activity.

Deletion of Postn in the MMTV-PyMT mouse model also results in normal tumor initiation and progression [22]. In addition, Postn-null tumor cells derived from Py-MT-expressing mice are able to grow efficiently in both wild-type and Postn-null mammary glands when orthotopically transplanted in Rag2−/− mice [22]. This supports the observations that primary tumors grow well in both wild-type and Postn-null mammary gland environments. This is in agreement with our findings showing that Neu + primary tumors develop and progress with similar kinetics in wild-type and Postn(−/−) mice. Interestingly, orthotopic or tail vein injection of Postn(−/−) Py-MT+ tumor cells results in very little lung colonization [22]. As tumor cells themselves do not express Postn, these data suggest that stroma-derived Postn is required for efficient colonization of secondary sites. Similarly, we find that subQ injection of wild-type Py-MT+ cells results in very poor growth in Postn-null mice, suggesting that Postn is required in the microenvironment for efficient tumor growth at secondary sites. Although a direct comparison between the different models is difficult, our study and the data by Malanchi *et al*. [22] support a role for Postn in the establishment and growth of mammary tumors at secondary sites.

As previously reported [22], we also did not find any differences in the ability of Postn(−/−) primary tumor cells to form tumorspheres or to modulate the canonical Wnt pathway (see Figure S4 in Additional file 5). One possibility is that the requirement of Postn is critical only at secondary sites for the expansion of cell subpopulations through a Wnt-dependent system [22]. However, our data suggest that restoring high levels of NICD or the activation of an AR-dependent system is sufficient to bypass the Postn requirement at secondary sites.

Interestingly, Postn has been shown to stimulate FAK and Akt activation downstream of integrin receptors under various conditions, contributing to increased invasion, angiogenesis and survival [12,13,15-17]. Our analysis shows that tumors derived from Postn-null animals display reduced levels of active FAK, supporting a role for Postn in their activation *in vivo*. However, no changes in phospho-Akt were observed, suggesting that additional pathways can maintain Akt activity. This is in marked contrast to what has been reported for pancreatic cancer cells [16]. Interestingly, overexpression of Postn in 293 cells has been reported to increase angiogenesis through FAK activation and vascular endothelial growth factor (VEGF) production [20]. However, no differences were observed in the number and size of CD31-positive blood vessels in tumors derived from Postn-null animals, suggesting that in this model, tumor angiogenesis can proceed without Postn.

Our data show that the loss of Postn results in reduced Notch activity and Hey1 transcription. Previous studies have shown that Hey1 and Hey1L can modulate AR transcriptional activity [72,73]. Similarly, expression of active Notch was found to downregulate AR activity in prostate cancer cells. Supporting these observations, we find that an approximate 50% decrease in Hey1 levels are accompanied with an increase in AR activity and target gene transcription. Interestingly, a 2-fold increase in AR protein levels was also observed. Whether this is also a Notch-regulated process in breast cancer cells remains to be investigated.

In mammary tumors from Postn-null mice, we have observed an increase in AR levels and an upregulation of its target genes PIP and Abp. The role of AR in hormone-dependent cancers is well documented [24]. However, the AR has recently received much attention as a novel therapeutic target in breast cancer [24-26] and a role for AR activation in a proportion of HER2+ cancers has been reported [26,28-30]. Recently, a large proportion of AR-positive molecular apocrine breast cancers have been shown to overexpress HER2 or PIP, an AR target gene [74]. Although PIP-null mice show no overt phenotype [75], PIP has been shown to be highly expressed in Luminal A and HER2 subtypes [71]. Silencing of PIP in T47D cells lead to a repression of MYC and decreased proliferation. In addition, decreased FAK, ERK and Akt activation were also observed. This supports other data that demonstrated a role for PIP in cell invasion [69]. Furthermore, a feed-forward loop between AR and PIP has been established [70]. These studies showed that PIP can induce AR expression and activity by stimulating its nuclear translocation. Similarly, we find that loss of Postn stimulates AR activity and target gene expression in primary tumors. Whether the establishment of a feed-forward loop in mammary tumors also follows the initial AR activation remains to be investigated. Nevertheless, our results show that deletion of Postn and upregulation of PIP is sufficient to overcome the loss of Postn *in vivo*, suggesting that PIP is a major effector of the AR response. How PIP mediates its effects on growth and invasion remains to be uncovered.

## Conclusions

Overall, our results show that Postn is not required for mammary gland development and ErbB2-driven tumorigenesis. However, deletion of Postn results in decreased Notch activity and an upregulation of AR levels that confers an apocrine-like subtype (Figure 7). We find that the growth defect of mammary tumor cells at heterotopic sites in a Postn-deficient environment can be rescued by NICD or PIP expression.

## Additional files

Additional file 1: Table S1. Primer pairs used in this study. **Table S2.** Microarray data showing the relative levels of genes for which the expression was altered by at least 10-fold in Postn-null tumors. Results are the average fold change from two independent tumors for both wild-type and Postn(−/−). Additional file 2: Figure S1. Postn deletion does not impair mammary gland development. (A) Example of a genotyping run showing an absence of the wild-type allele in homozygote mutant mice. (B) Western blot analysis of whole mammary gland lysates showing Postn expression in wild-type and heterozygotes. Postn reactivity is lost in Postn(−/−) glands. Postn runs as 80-90 kDa glycosylated isoforms. Representative images of mammary gland whole mounts from nulliparous (C-E) and pregnant (F-H) FVB Postn^+/+^, Postn^+/−^, and Postn^−/−^ mice at 8 weeks and 14.5 days post coitum (dpc). No differences were observed in ductal outgrowth and arborization. (I-K) High magnification of panels shown in F to H. Anti-Postn immunohistochemistry showing Postn expression in wild-type mice (L and N). No reactivity was observed in Postn-null mammary glands (M and O). Postn reactivity was found exclusively in the stromal compartment lining the ducts (arrowhead). Some staining was also observed in the adipose tissue. Additional file 3: Figure S2. Deletion of Postn does not impair tumor initiation. Representative H&E staining of a paraffin-embedded whole mammary gland from Postn wild-type (A) or Postn(−/−) (B) females showing multiple hyperplastic foci at 4 months of age. (C and D) Magnification of the boxed area from A or B. (C) Quantitation of the average number of hyperplastic lesions per section of whole mammary gland in 4-month-old females. Additional file 4: Figure S3. Postn deletion does not impair tumor growth or angiogenesis. Frozen sections from wild-type mammary tumors were immunostained for CD31 (A) or Ki-67 (C) to detect newly formed blood vessels and proliferating cells, respectively. No differences were observed when compared to Postn(−/−) tumors (B and D). (E) Quantitation of Ki-67+ cells in three independent tumors. The proportion of Ki-67+ cells was calculated relative to the total number of nuclei in the field. At least 1,000 nuclei were counted. Additional file 5: Figure S4. Postn deletion does not impair β-catenin levels or signaling in tumor cells. (A) Western blot analysis showing total β-catenin levels from three independent tumors from each genotype. Representative of an anti-β-catenin IHC on a wild-type (B) or Postn-null tumor (C) showing cytosolic and nuclear localization. No differences were observed between the two genotypes. (D) TOP-Flash Luciferase assays on Met-1 cells expressing Postn or active Notch (NICD). No further activation was observed in the presence of Postn or NICD when compared to vector control (pPLCX). Additional file 6: Figure S5. (A) Q-PCR expression analysis of Notch mRNA in NICD- and ΔNLS- transfected cultures. Exogenous NICD and ΔNLS were found to be overexpressed 18- and 4-fold, respectively. (B) Western blot analysis of AR protein levels in subQ tumors from various Met-1 pools injected into wild-type or Postn-null mice. Additional file 7: Figure S6. Loss of Postn does not affect tumorsphere formation. Representative photomicrograph of tumorsphere cultures derived from wild-type (A) and Postn-null (B) tumors. (C) Quantitation of primary and secondary tumorspheres from both genotypes after 7 days in cultures. No differences were observed in the number and size of the spheres between wild-type and Postn-null tumors. Spheres were counted from five independent tumors and tumorsphere cultures could be established from all tumor samples.

## Abbreviations

Abp: androgen-binding proteins
AR: androgen receptor
DMEM: Dulbecco’s modified Eagle’s medium
ECM: extracellular matrix
EGFR: epidermal growth factor receptor
ER: estrogen receptor
FAK: focal adhesion kinase
FCS: fetal calf serum
H&E: hematoxylin and eosin
HRP: horseradish peroxidase
MMTV: mouse mammary tumor virus
OSF-1: osteoblast-specific factor-2
qRT-PCR: quantitative real-time polymerase chain reaction
PIP: prolactin-induced protein
Postn: periostin
PVDF: polyvinylidene fluoride
PyMT: polyoma middle T antigen
RTKs: receptor tyrosine kinases
SDS: sodium dodecyl sulfate
TMA: tissue microarrays
VEGF: vascular endothelial growth factor
