# Supplementary figures and images for: Loss of periostin/OSF-2 in ErbB2/Neu-driven tumors results in androgen receptor-positive molecular apocrine-like tumors with reduced Notch1 activity
Source: Breast Cancer Res. 2015 Jan 16;17(1):7. doi: 10.1186/s13058-014-0513-8 (PMC4355979; doi:10.1186/s13058-014-0513-8)

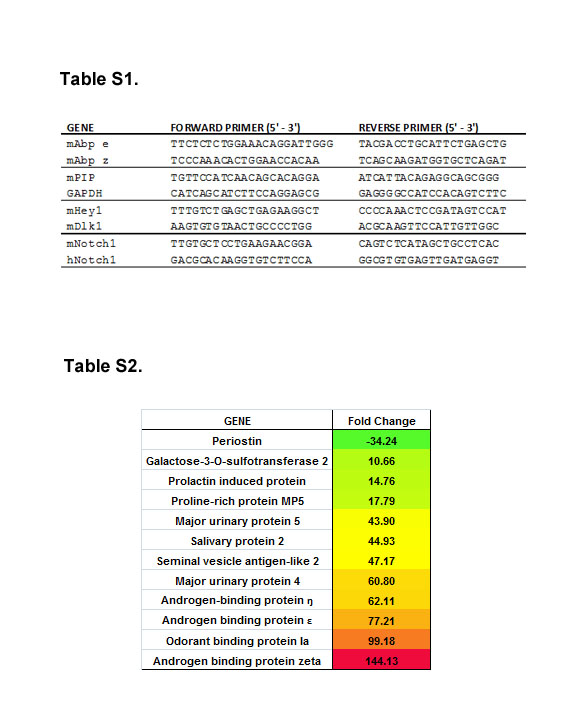

Supplement: Additional file 1: Table S1. — Primer pairs used in this study. Table S2. Microarray data showing the relative levels of genes for which the expression was altered by at least 10-fold in Postn-null tumors. Results are the average fold change from two independent tumors for both wild-type and Postn(−/−). [file 13058_2014_513_MOESM1_ESM.jpeg]

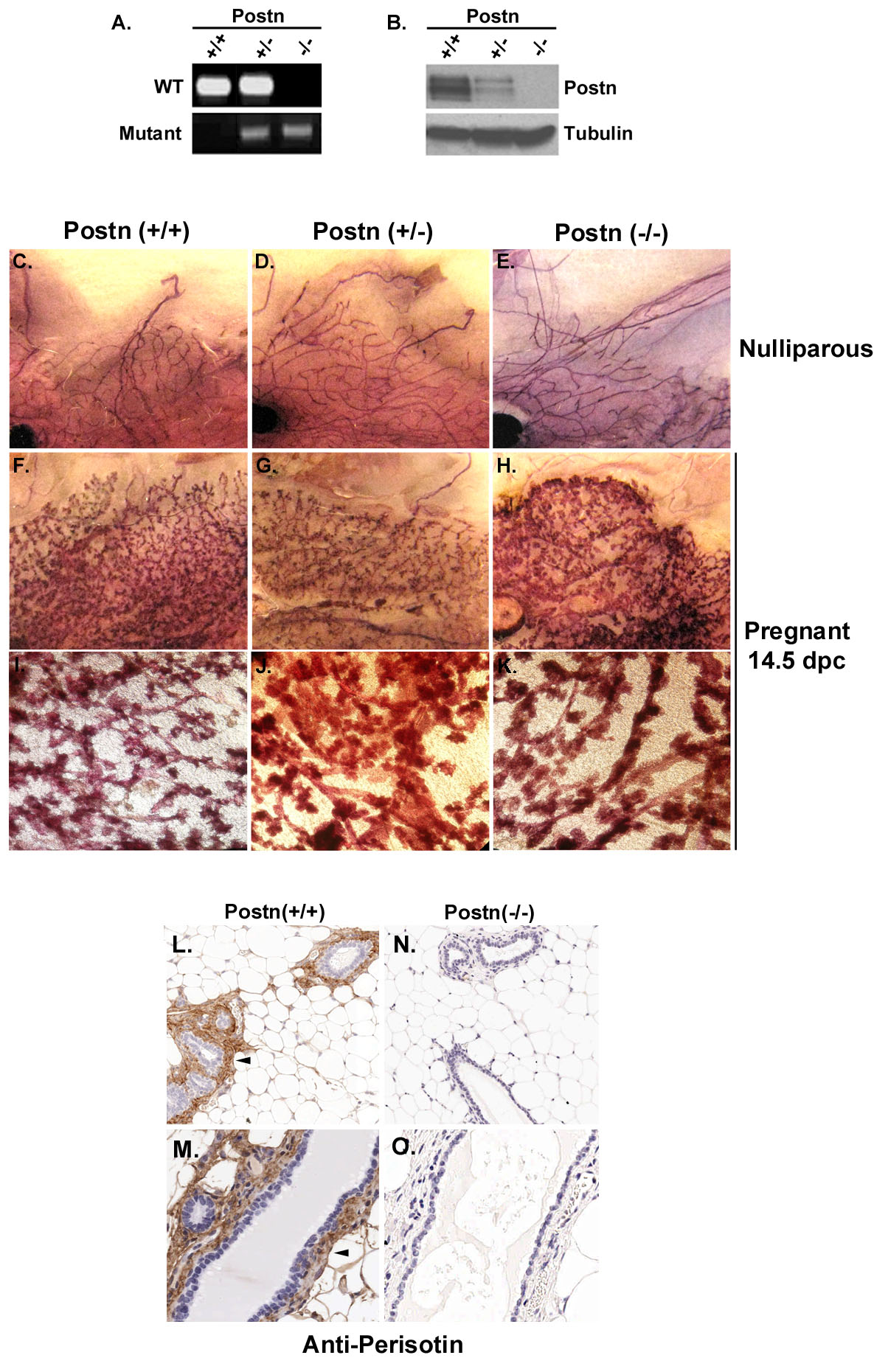

Supplement: Additional file 2: Figure S1. — Postn deletion does not impair mammary gland development. (A) Example of a genotyping run showing an absence of the wild-type allele in homozygote mutant mice. (B) Western blot analysis of whole mammary gland lysates showing Postn expression in wild-type and heterozygotes. Postn reactivity is lost in Postn(−/−) glands. Postn runs as 80-90 kDa glycosylated isoforms. Representative images of mammary gland whole mounts from nulliparous (C-E) and pregnant (F-H) FVB Postn+/+, Postn+/−, and Postn−/− mice at 8 weeks and 14.5 days post coitum (dpc). No differences were observed in ductal outgrowth and arborization. (I-K) High magnification of panels shown in F to H. Anti-Postn immunohistochemistry showing Postn expression in wild-type mice (L and N). No reactivity was observed in Postn-null mammary glands (M and O). Postn reactivity was found exclusively in the stromal compartment lining the ducts (arrowhead). Some staining was also observed in the adipose tissue. [file 13058_2014_513_MOESM2_ESM.jpeg]

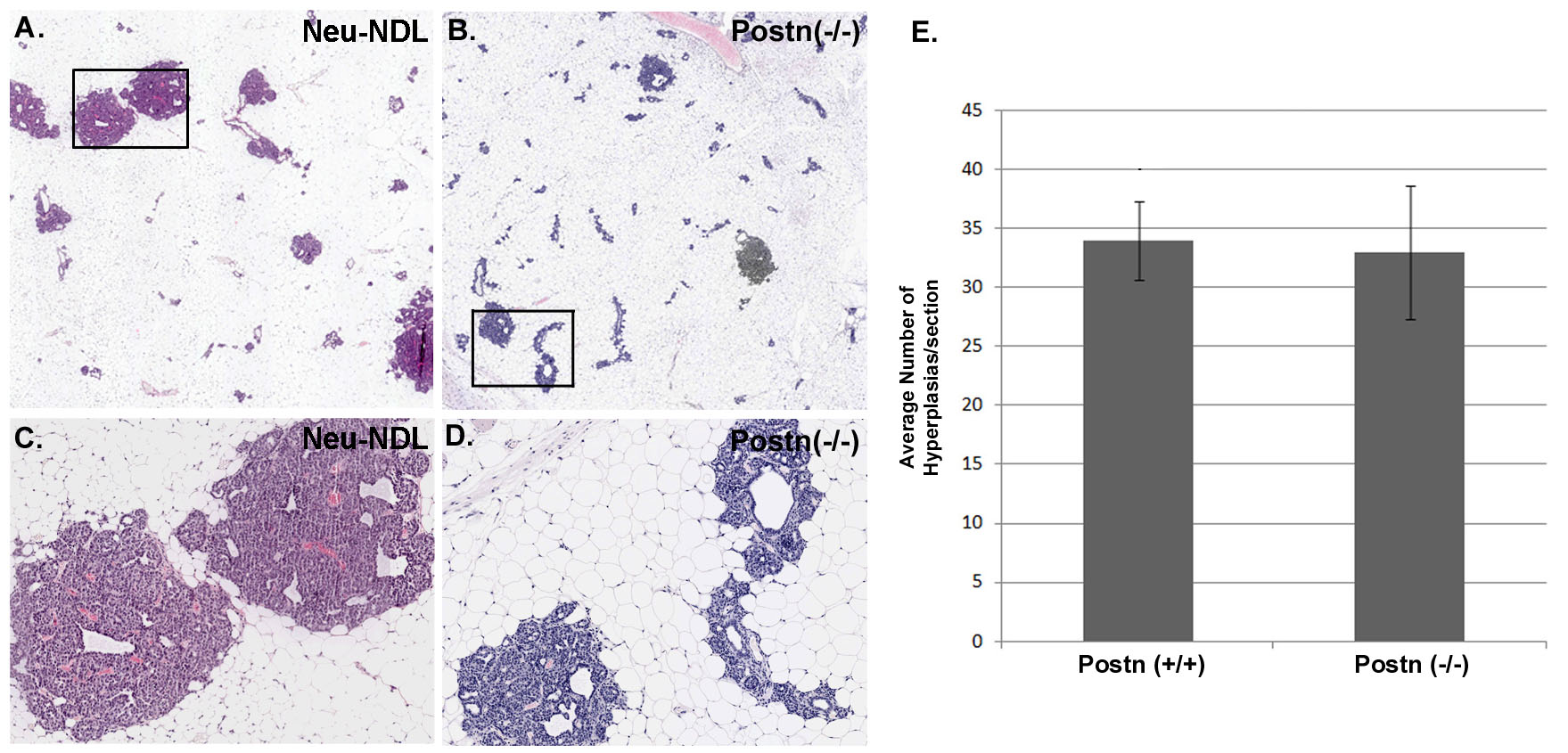

Supplement: Additional file 3: Figure S2. — Deletion of Postn does not impair tumor initiation. Representative H&E staining of a paraffin-embedded whole mammary gland from Postn wild-type (A) or Postn(−/−) (B) females showing multiple hyperplastic foci at 4 months of age. (C and D) Magnification of the boxed area from A or B. (C) Quantitation of the average number of hyperplastic lesions per section of whole mammary gland in 4-month-old females. [file 13058_2014_513_MOESM3_ESM.jpeg]

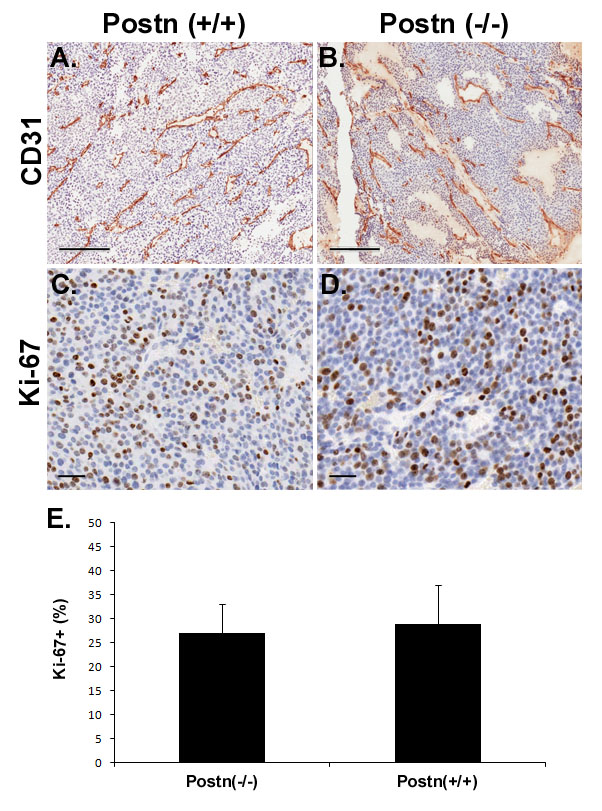

Supplement: Additional file 4: Figure S3. — Postn deletion does not impair tumor growth or angiogenesis. Frozen sections from wild-type mammary tumors were immunostained for CD31 (A) or Ki-67 (C) to detect newly formed blood vessels and proliferating cells, respectively. No differences were observed when compared to Postn(−/−) tumors (B and D). (E) Quantitation of Ki-67+ cells in three independent tumors. The proportion of Ki-67+ cells was calculated relative to the total number of nuclei in the field. At least 1,000 nuclei were counted. [file 13058_2014_513_MOESM4_ESM.jpeg]

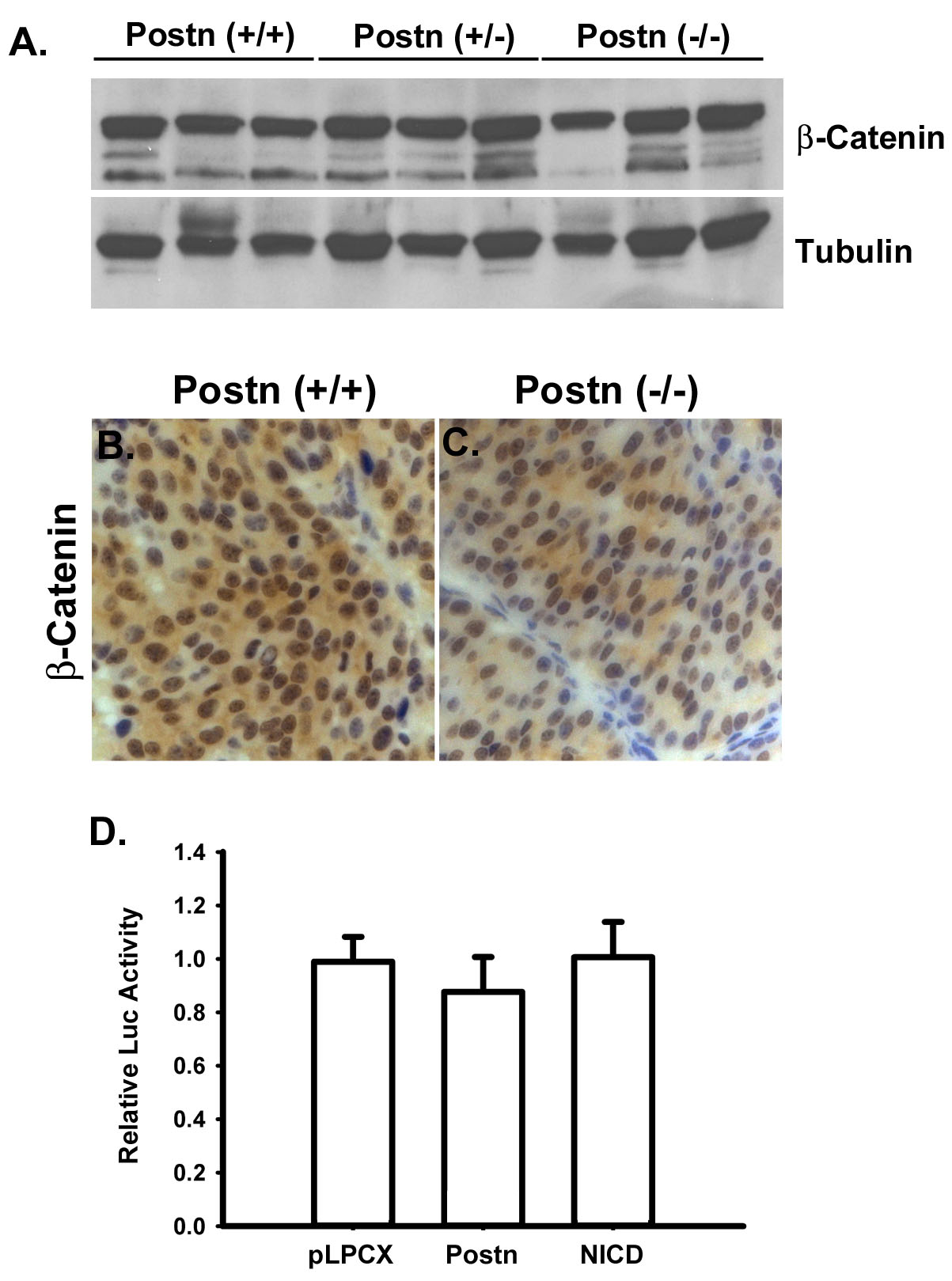

Supplement: Additional file 5: Figure S4. — Postn deletion does not impair β-catenin levels or signaling in tumor cells. (A) Western blot analysis showing total β-catenin levels from three independent tumors from each genotype. Representative of an anti-β-catenin IHC on a wild-type (B) or Postn-null tumor (C) showing cytosolic and nuclear localization. No differences were observed between the two genotypes. (D) TOP-Flash Luciferase assays on Met-1 cells expressing Postn or active Notch (NICD). No further activation was observed in the presence of Postn or NICD when compared to vector control (pPLCX). [file 13058_2014_513_MOESM5_ESM.jpeg]

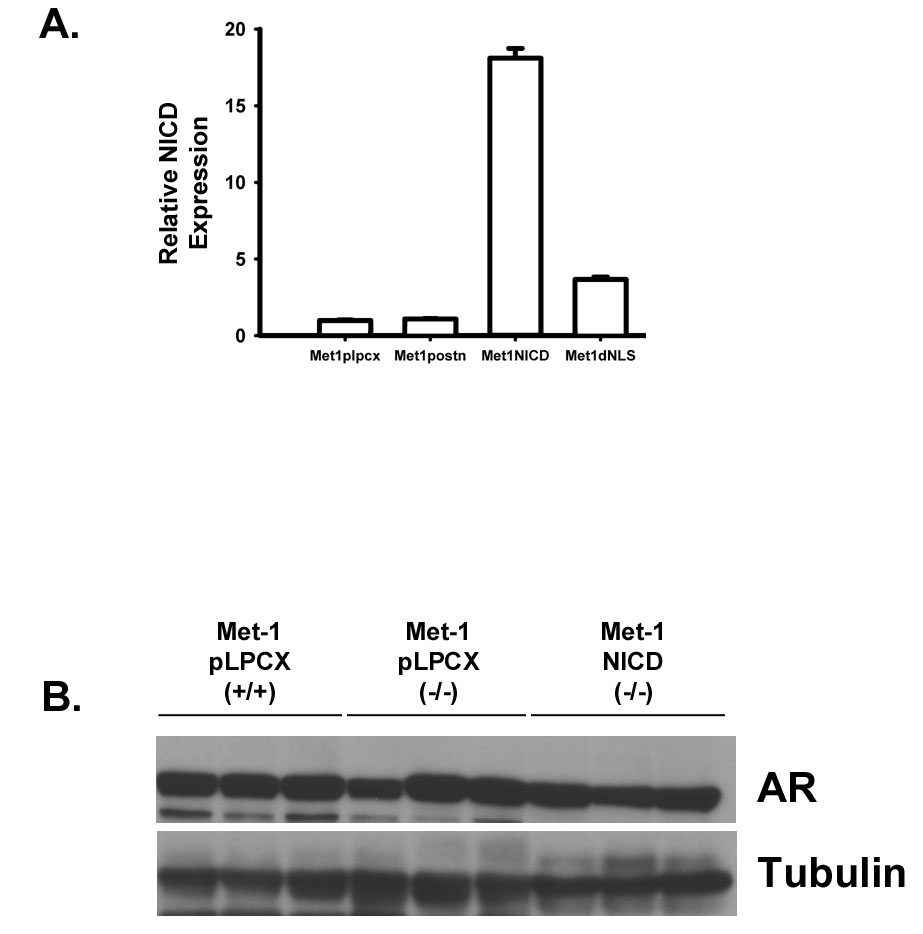

Supplement: Additional file 6: Figure S5. — (A) Q-PCR expression analysis of Notch mRNA in NICD- and ΔNLS- transfected cultures. Exogenous NICD and ΔNLS were found to be overexpressed 18- and 4-fold, respectively. (B) Western blot analysis of AR protein levels in subQ tumors from various Met-1 pools injected into wild-type or Postn-null mice. [file 13058_2014_513_MOESM6_ESM.jpeg]

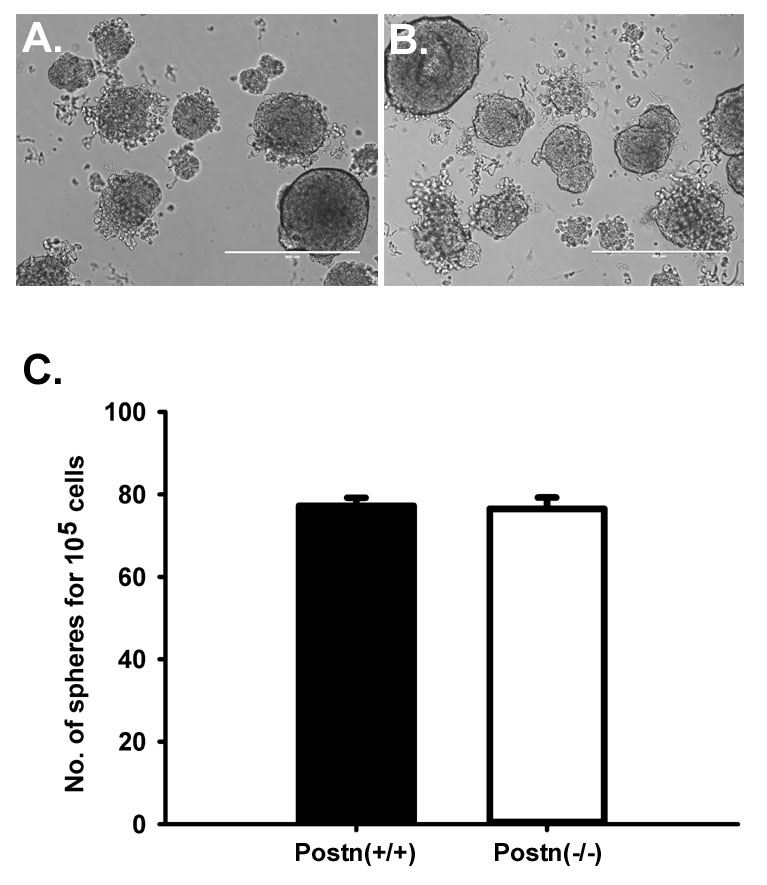

Supplement: Additional file 7: Figure S6. — Loss of Postn does not affect tumorsphere formation. Representative photomicrograph of tumorsphere cultures derived from wild-type (A) and Postn-null (B) tumors. (C) Quantitation of primary and secondary tumorspheres from both genotypes after 7 days in cultures. No differences were observed in the number and size of the spheres between wild-type and Postn-null tumors. Spheres were counted from five independent tumors and tumorsphere cultures could be established from all tumor samples. [file 13058_2014_513_MOESM7_ESM.jpeg]
